# Comparative analysis of miRNA expression in Yili horses pre- and post-5000-m race

**DOI:** 10.3389/fgene.2025.1676558

**Published:** 2025-10-01

**Authors:** Shikun Ma, Wanlu Ren, Zexu Li, Luling Li, Ran Wang, Yi Su, Qiuping Huang, Shan Dehaxi, Jianwen Wang

**Affiliations:** ^1^ College of Animal Science, Xinjiang Agricultural University, Urumqi, China; ^2^ Xinjiang Key Laboratory of Equine Breeding and Exercise Physiology, Urumqi, China

**Keywords:** Yili horse, athletic performance, epigenetics, miRNA, candidatetarget gene, signal transduction pathway

## Abstract

Equine athletic performance is modulated by both genetic and epigenetic mechanisms. As dynamic regulators of gene expression, MicroRNAs (miRNAs) play a central role in the physiological response to exercise-induced stress18. This study focused on the top three elite Yili horses from a 5000-m race, collecting peripheral blood samples pre-race (group B) and post-race (group A). A longitudinal comparative analysis integrating miRNA omics profiling and target gene functional enrichment was performed. Nineteen miRNAs exhibited significant differential expression (10 upregulated, 9 downregulated), with their associated genes primarily implicated in small GTPase-mediated signal transduction, supramolecular complex, and molecular function regulator. Pathway enrichment analysis identified significant associations with Rap1, Ras, and Phospholipase D signaling pathways. These findings suggest that miRNA-mediated regulation may contribute to exercise adaptation by modulating cytoskeletal remodeling and metabolic reprogramming. The study elucidates epigenetic regulatory features underpinning the 5000-m race stress response in Yili horses via omics technology, offering novel insights into the molecular basis of exercise adaptation and establishing quantifiable miRNA markers to inform early-stage equine breeding strategies.

## 1 Introduction

The Yili horse, originating from the Yili Kazakh Autonomous Prefecture in the Xinjiang Uygur Autonomous Region of China, was developed during the last century by crossing native Kazakh mares with stallions of Orlov, Budyonny, and Russian Don (Wang et al., 2024). The speed of the Yili horse is among the best of Chinese horse breeds, yet there remains a gap compared to major international racing breeds such as the Thoroughbred. Therefore, analyzing the exercise regulation mechanisms of the Yili horse to improve its racing performance is of great significance for enhancing the overall level of horse racing in China. Genetic factors constitute primary regulators of equine athletic traits, and elucidating their molecular basis is essential for precision breeding. Conventional genetic analyses have established moderate to high heritability for exercise-related phenotypes, alongside marked genetic divergence across breeds (Wang C. K. et al., 2025; Welker et al., 2018). Existing studies have identified some molecular markers related to horse movement or speed, such as *GSDMC* gene (Liu et al., 2025). Nonetheless, the observed phenotypic plasticity implies a substantial contribution from epigenetic modulation.

Emerging evidence highlights the capacity of epigenetic processes-specifically DNA methylation, histone modification, and non-coding RNAs-to respond dynamically to environmental inputs such as training, thereby fine-tuning gene expression in support of exercise adaptation (Cappelli et al., 2024). Among these regulators, miRNAs have garnered significant attention for their role in post-transcriptional gene regulation within exercise-induced stress responses (Li et al., 2025; Zhang et al., 2025).

miRNA is a class of endogenous non-coding small RNAs approximately 22 nucleotides in length, exhibiting high sequence conservation and widespread presence in eukaryotic cells (Wang C. et al., 2025). Its biogenesis initiates with RNA polymerase II-mediated transcription of precursor genes, yielding pri-miRNA, which is subsequently processed by the Drosha-DGCR8 complex into pre-miRNA with a hairpin structure. This intermediate is then exported to the cytoplasm via Exportin-5 and further cleaved by Dicer to generate a mature miRNA duplex. The mature strand associates with the RNA-induced silencing complex and binds to the 3′UTR of target mRNAs through sequence complementarity, resulting in either mRNA degradation or translational repression, thereby negatively regulating gene expression (Hussein, 2012). Accumulating evidence indicates that miRNA participates in the regulation of cellular processes including proliferation, apoptosis, energy metabolism, immune modulation, and tissue repair, and mediates molecular adaptations in response to exercise stimuli (Wilczynska and Bushell, 2015). At the same time, miRNAs are ideal non-invasive biomarkers for motor adaptation monitoring due to their stability in body fluids, tissue-specific expression patterns, and dynamic response to stimuli. In a study on horses (Herkenhoff, 2025), miRNAs are deeply involved in the adaptive regulation of athletic performance, such as muscle development, energy metabolism, and post-exercise inflammatory responses, by regulating gene expression (Bou et al., 2022; Wang T. L. et al., 2025). In terms of diseases, specific miRNA expression profiles are closely related to the occurrence and development of various common diseases such as osteoarthritis, respiratory diseases, and laminitis, making them promising new diagnostic biomarkers and therapeutic targets. However, there is a serious lack of research on the mechanism of exercise adaptation, especially the lack of data on local varieties in China, which restricts the application breakthrough of exercise epigenetics.

As a key modulator of gene expression, miRNA exhibits dynamic responsiveness to exercise-induced physiological stress. Acute physical activity rapidly alters the expression of myogenic miRNAs (myomiRs), including *miR-1* and *miR-133* families, which regulate mitochondrial biogenesis and muscle remodeling by targeting genes such as *HDAC4* and *NRF1* (Safdar et al., 2009; Zacharewicz et al., 2013). Endurance exercise downregulates *miR-23*, thereby lifting repression on *PGC-1α* (Safdar et al., 2009), and enhancing oxidative metabolic adaptation. Expression of *miR-206*, *miR-133a*, and *miR-133b* contributes to skeletal muscle differentiation and may influence muscle growth (Kirby and McCarthy, 2013). *miR-146a-5p* is upregulated by inflammatory stimuli and attenuates the NF-κB pathway by targeting proinflammatory transcripts such as *IRAK1* and *TRAF6*, thus modulating cytokine signaling and nociceptive processes (Li et al., 2011; Iyer et al., 2012; Roos et al., 2016). Collectively, current evidence suggests that miRNAs act as molecular regulators of exercise adaptation; however, research in this area remains relatively limited in horses (Ding et al., 2025; Yuan et al., 2025), and the mechanisms through which miRNAs regulate exercise responses in equines are still not fully unclear. By integrating whole-transcriptomic profiling with functional enrichment analysis, the present study delineates the exercise-responsive miRNA dynamics in Yili horses, advancing the understanding of epigenetic regulation in equine exercise physiology and informing the development of early-stage, quantifiable markers for breeding selection.

## 2 Materials and methods

### 2.1 Horse blood collection

**FIGURE 1:**
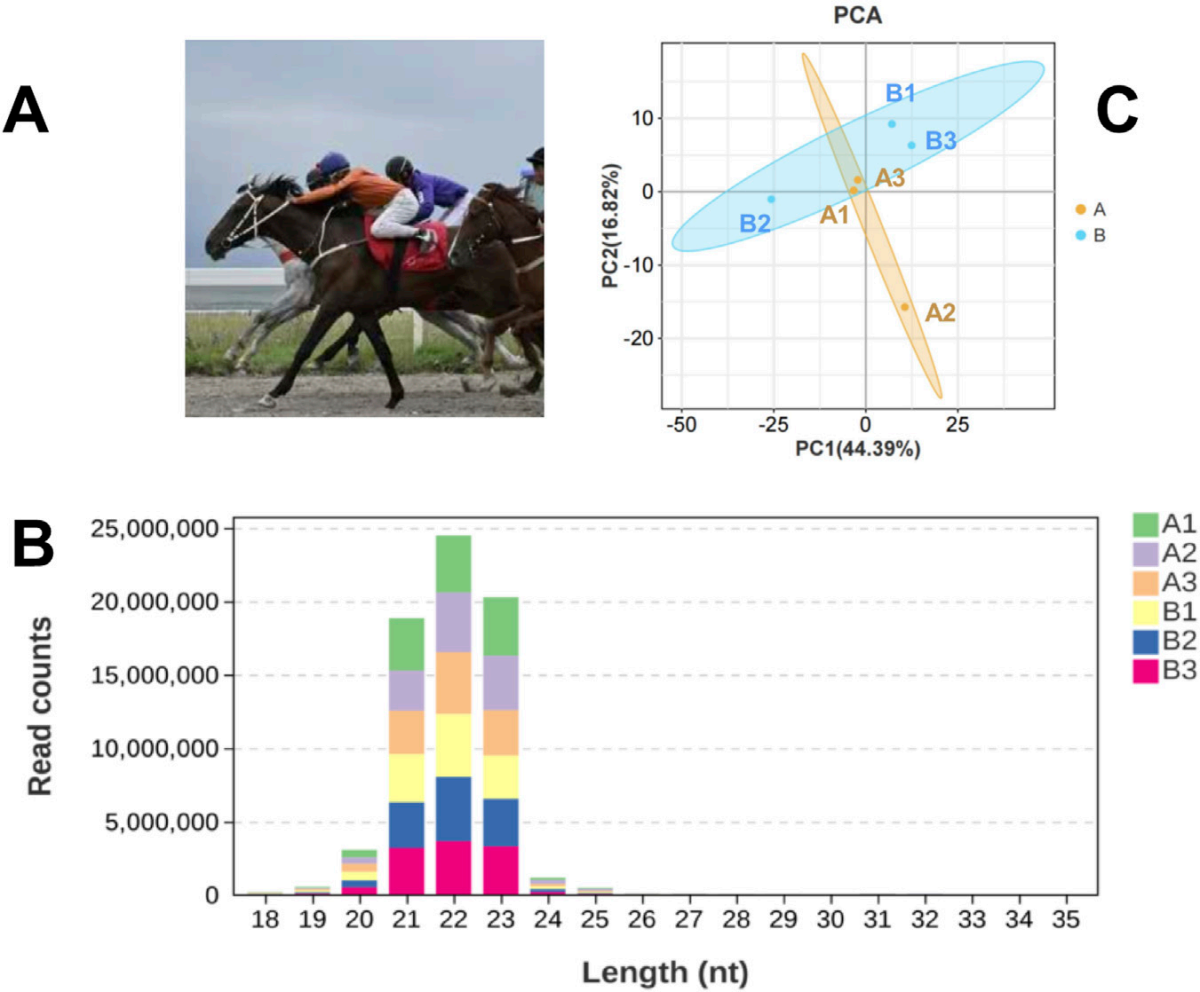
Basic Information of the Experiment. **(A)** Yili horses in competition; **(B)** Statistical Analysis of miRNA Length Distribution in Groups A and B Libraries; **(C)** Principal component analysis (PCA) of samples.

We organized a 5000-m race with 24 Yili horses (Figure 1A), all of which were over 3 years old. At 20:00 on the day before the race, jugular venous blood samples were collected from all participating horses. Within 5 min after the race, samples were obtained from the top three performers. Examination revealed that these three horses were all four-year-old stallions. The pre-race blood samples of these horses were retrieved and paired with their post-race samples for subsequent analysis. The race times of these three horses ranged from 5′23″704 to 5′41″339. All samples were preserved in liquid nitrogen. Prior to the race, horses underwent conditioning regimens consistent with those used for thoroughbreds, Horses are subjected to similar basic endurance training (3-4 times a week, 5–10 km cantering) as well as speed training (1-2 times a week, 800–1000 m intermittent sprints) at the owner’s place. The race track is a standard sand track, and all horses are placed in the same stable environment and fed a standard ration 48 h before the race. Pedigree verification and health assessments, including lameness examinations, were conducted by veterinarians in accordance with pre-race protocols. All blood samples from the horses were collected by the same skilled veterinarian using vacuum tubes. To minimize stress on the animals, each sample did not exceed 5 mL, and the entire collection process was completed within 1 min.

### 2.2 RNA extraction and quality analysis

Total RNA was isolated from the blood samples of groups A and B using the TRIzol kit (Invitrogen, Carlsbad, CA, United States). RNA degradation and contamination were assessed via 1% agarose gel electrophoresis. RNA yield and purity were quantified using a NanoPhotometer® spectrophotometer, while concentrations were determined with the Qubit® RNA Assay Kit on a Qubit® 2.0 Fluorometer (Life Technologies, Carlsbad, CA, United States). RNA integrity was evaluated with the Nano 6000 Assay Kit on the Agilent Bioanalyzer 2,100 system (Agilent Technologies, Santa Clara, CA, United States), and samples with RIN values exceeding 7.0 were considered suitable. Small RNA fractions were subsequently utilized for RNA-seq and qRT-PCR analyses.

### 2.3 Small RNA sequencing and data analysis

#### 2.3.1 Library preparation and small RNA sequencing

Total RNA was subjected to library construction in accordance with the NEB Next Ultra Small RNA Sample Library Preparation Kit protocol (New England Biolabs, Ipswich, MA, United States) designed for Illumina sequencing. Only libraries meeting quality standards proceeded to high-throughput sequencing. PCR-amplified products were separated and purified via 8% polyacrylamide gel electrophoresis (100 V, 80 min), and 140–160 bp DNA fragments were recovered using 8 μL elution buffer. Library quality was assessed with the Agilent Bioanalyzer 2,100 system employing a DNA high sensitivity chip. Sequencing was performed on the Illumina HiSeq X Ten platform (New England Biolabs, Ipswich, MA, United States), generating 50-nucleotide single-end (SE) read.

#### 2.3.2 Data processing

Cutadapt (v1.2.2) (http://code.google.com/p/cutadapt/) was employed to remove the 3′adapter sequences, followed by selection of small RNAs (sRNAs) ranging from 18 to 30 nt for subsequent analysis. Low-quality reads (Phred score <30) were excluded using the FASTQ quality filter provided by the FASTX-Toolkit v0.0.13.2 (http://hannonlab.cshl.edu/fastx_toolkit/). High-quality reads were then aligned to the EquCab 3.0 horse genome using Bowtie v1.0.1. Known miRNAs were annotated based on miREvo (v1.1), while novel miRNA candidates were identified via miRDeep2 (v0.0.5).

Expression levels of both annotated and novel miRNAs were quantified using TPM (transcripts per million), with normalization calculated as:

$$\text{TPM}=\left(\text{aligned read count }\times \;1,000,000\right)\;/\text{total reads}$$

#### 2.3.3 Identification of differentially expressed miRNAs and functional enrichment

We employed DESeq2 (v1.24.0) to conduct the differential expression analysis between the two groups of samples (group A post-race and group B pre-race), with key parameters rigorously applied to ensure robust results: the adjusted P-value threshold was set to<0.05 using the Benjamini-Hochberg method. For target gene prediction of the identified miRNAs, we utilized two complementary computational tools-miRanda (v3.3a) with stringent thresholds of score≥140 and binding energy ≤ −10 kcal/mol to prioritize high-affinity interactions, and RNAhybrid (v2.0) requiring P-value ≤0.05 and minimum free energy ≤ −10 kcal/mol to validate thermodynamic stability-adhering to a strict selection criterion where only genes predicted by both algorithms (intersecting results) were retained for subsequent functional analysis.

Gene Ontology (GO) annotation and Kyoto Encyclopedia of Genes and Genomes (KEGG) pathway enrichment analysis of the target genes were performed using the ClusterProfiler (v3.8.1). Statistical significance for enriched functional categories and pathways was determined by a corrected P-value threshold of padj< 0.05, as indicated in the bioinformatics workflow parameters.

### 2.4 Validation of miRNA expression by qRT-PCR

Total RNA was isolated from blood samples collected from Yili horses pre-race (Group B) and post-race (Group A) using standard extraction procedures, followed by reverse transcription with the ReverTra Ace® qPCR RT Kit (TOYOBO, FSQ-101). The 20 μL reverse transcription reaction included 4 μL of 5×RT Buffer, 1 μL Enzyme Mix, 0.5 μL Primer Mix, 2 μg total RNA, and RNase-free water. The reaction was conducted at 37 °C for 15 min and 98 °C for 5 min using a Thermo Veriti PCR instrument. Differentially expressed miRNAs (*miR-128*, *miR-143*, *miR-148a*, *miR-486-3p*, and *let-7g*) were randomly selected for analysis, with specific forward primers designed accordingly (primer sequences provided in Table 1). U6 snRNA served as the internal control, using forward primer CTCGCTTCGGCAGCACA and reverse primer AAC​GCT​CAC​GAA​TTT​GCG​T. qPCR amplification was carried out in a 10 μL system comprising 5 μL SYBR® Green Realtime PCR Master Mix (TOYOBO, QPK-201), 0.4 μL of each forward and reverse primer (10 μM), 0.8 μL cDNA template, and Nuclease-Free Water. The amplification was performed using a TL-988 fluorescence qPCR instrument (Tianlong, Xi’an, China) under the following cycling conditions: initial denaturation at 95 °C for 30 s, followed by 40 cycles of denaturation under 95 °C for 15 s and annealing/extension under 60 °C for 30 s, and a final melting curve analysis from 65 °C to 95 °C. Relative expression levels were determined using the 2^-△△Ct^ method, and statistical comparisons were performed using a paired t-test in SPSS 26, with *P* < 0.05 indicating significance. Each sample was processed in triplicate, and Ct value variation remained below 5%.

**TABLE 1:** RT-qPCR primer sequences corresponding to each miRNA.

Primer name	Primer sequence
*eca-let-7g*	TGA​GGT​AGT​AGT​TTG​TAC​AGT​T
*eca-miR-128*	TCA​CAG​TGA​ACC​GGT​CTC​TTT
*eca-miR-148a*	TCA​GTG​CAC​TAC​AGA​ACT​TTG​T
*eca-miR-143*	TGA​GAT​GAA​GCA​CTG​TAG​CTC
*eca-miR-486-3p*	CGG​GGC​AGC​TCA​GTA​CAG​GAT
U6-F	CTCGCTTCGGCAGCACA
U6-R	AAC​GCT​TCA​CGA​ATT​TGC​GT

## 3 Results

### 3.1 Transcriptome sequencing

An average of 0.59 G of sequencing data was generated per sample, with Q30 values exceeding 97.72% and GC content ranging from 53% to 55%. Following quality filtering, over 99.27% of raw reads were retained. Clean reads were predominantly distributed between 18 and 30 nt, consistent with the size profile of animal sRNAs. Notably, miRNAs were mainly concentrated within the 21-24 nt range (Figure 1B). Approximately 98% of sRNAs were successfully aligned to the reference genome. Analysis of known miRNAs using the miRBase database identified 318 miRNAs, while novel miRNA prediction yielded an additional 129 candidates (detailed in Table 2–5).

**TABLE 2:** Sequencing data.

Sample	Raw reads	Bases	Clean reads	Q30 (%)	GC content
A1	12,592,927	0.63G	12,521,184 (99.43%)	98.36	55.03
A2	11,792,521	0.59G	11,706,781 (99.27%)	97.92	53.94
A3	11,405,060	0.57G	11,332,232 (99.36%)	98.14	54.20
B1	11,802,789	0.59G	11,725,997 (99.35%)	98.30	53.71
B2	11,749,085	0.59G	11,680,301 (99.41%)	98.17	53.38
B3	11,490,798	0.57G	11,418,748 (99.37%)	97.72	53.70

**TABLE 3:** Summary of sRNA types and quantities.

Sample	Total reads	Total bases (bp)	Uniq reads	Uniq bases (bp)	Mapped sRNA
A1	12,428,825	273,606,051	74,644	1,730,302	11,357,001 (98.07%)
A2	11,653,738	258,855,654	98,406	2,334,124	11,413,287 (98.13%)
A3	11,244,256	246,827,493	67,570	1,530,328	11,028,498 (97.80%)
B1	11,580,587	253,910,266	83,283	1,907,785	12,179,233 (97.99%)
B2	11,630,358	255,884,615	73,531	1,693,751	11,320,672 (97.14%)
B3	11,276,337	247,982,807	84,225	1,936,922	11,013,492 (97.95%)

**TABLE 4:** Summary of known miRNA comparisons for each sample.

Types	Total	A1	A2	A3	B1	B2	B3
Mapped mature	318	250	270	241	250	242	268
Mapped hairpin	359	280	295	273	282	268	305
Mapped uniq sRNA	12,533	1960	2,210	1859	2,107	2,157	2,240
Mapped total sRNA	41,822,066	7,396,765	6,409,997	7,033,455	7,212,255	7,117,430	6,652,164

**TABLE 5:** Summary of predicted novel miRNAs and comparisons with sRNAs for each sample.

Types	Total	A1	A2	A3	B1	B2	B3
Mapped mature	129	77	82	75	98	81	90
Mapped star	44	18	21	21	22	20	23
Mapped hairpin	136	84	88	85	102	86	98
Mapped uniq sRNA	1,617	244	278	240	313	259	283
Mapped total sRNA	51,945	5,945	10,882	7,541	9,756	9,931	7,890

Principal component analysis (PCA) (Figure 1C) revealed partial overlap between group A (orange) and group B (blue), with a discernible directional shift—samples in group A trending leftward and those in group B trending rightward. Although inter-group distinctions were present, complete separation was not achieved. The presence of broadly distributed non-differential miRNAs likely attenuated group-level divergence.

### 3.2 Differential miRNAs

**FIGURE 2:**
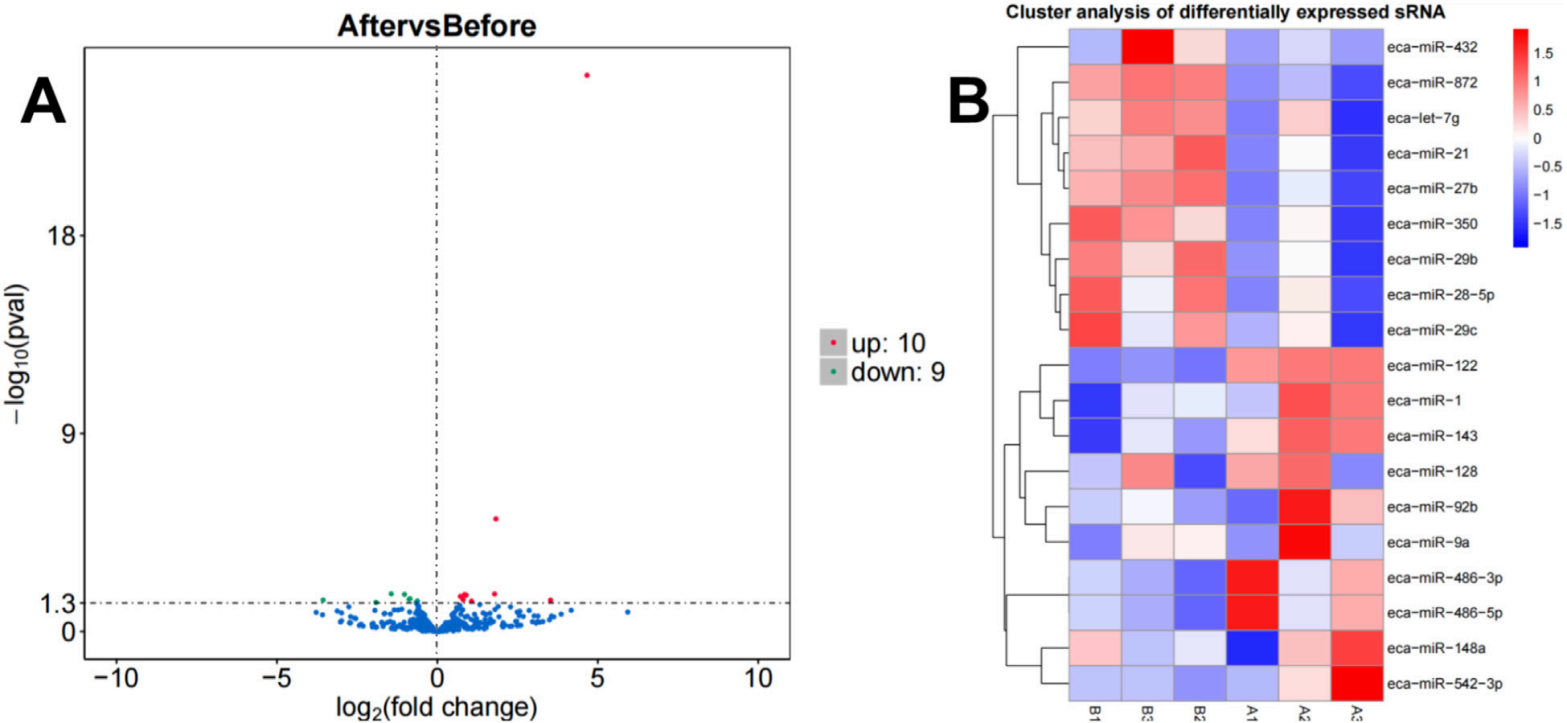
Volcano plot **(A)** and heatmap **(B)** illustrating miRNA expression differences between group B and group A.

A total of 19 differentially expressed miRNAs were identified under the criteria of P-value <0.05 and |log2FC| > 0. According to the volcano plot (Figure 2A), comparison between group A and group B revealed 10 upregulated and 9 downregulated miRNAs. Integrated with the clustering analysis shown in the heat map (Figure 2B), the upregulated miRNAs included *miR-1*, *miR-122*, *miR-128*, *miR-143*, *miR-148a*, *miR-486-3p*, *miR-486-5p*, *miR-542-3p*, *miR-92b*, and *miR-9a*, while the downregulated miRNAs comprised *let-7g*, *miR-21*, *miR-27b*, *miR-28-5p*, *miR-29b*, *miR-29c*, *miR-350*, *miR-432*, and *miR-872*.

### 3.3 Enrichment analysis

**FIGURE 3:**
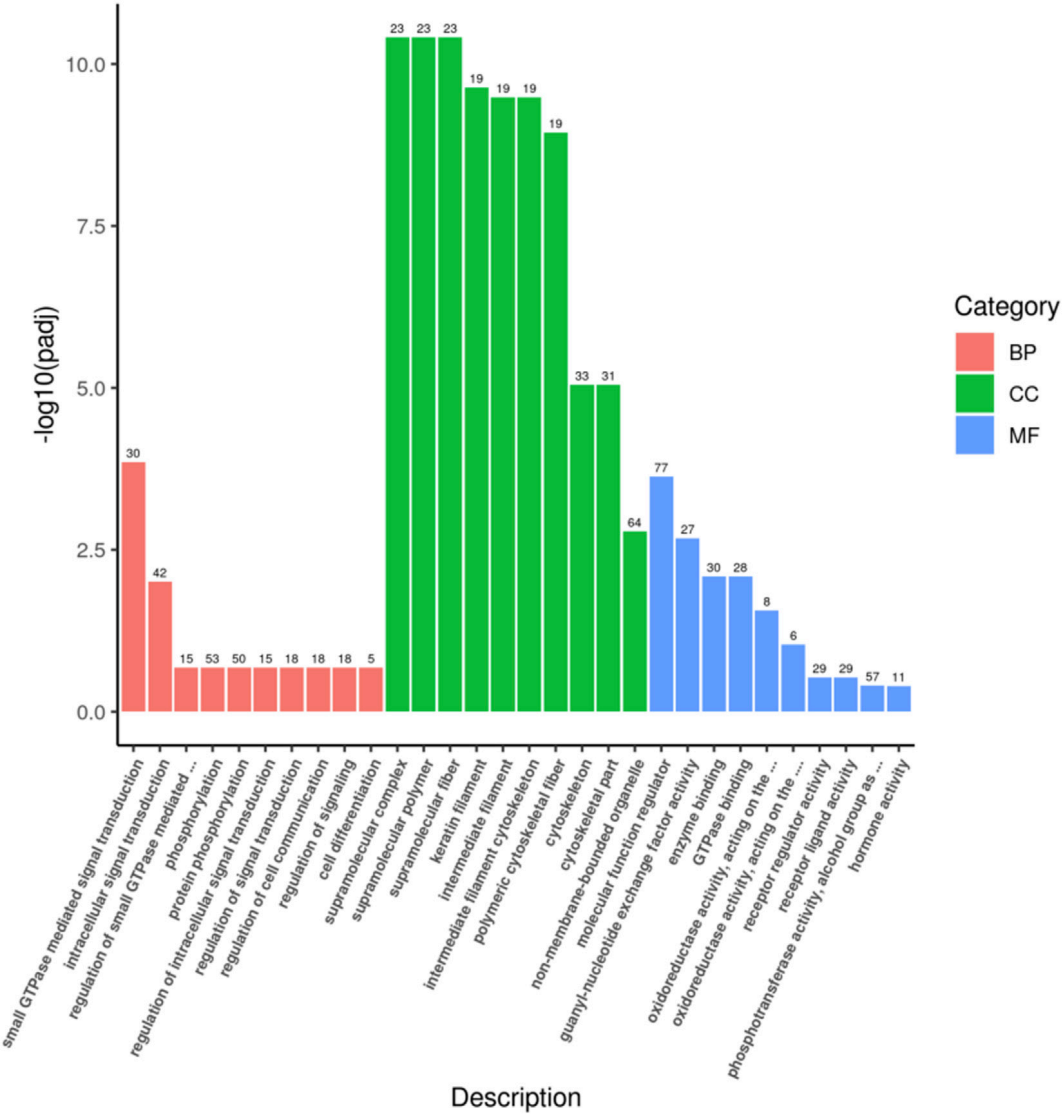
GO enrichment analysis histogram.

A total of 165 target genes were covered by GO functional annotations, and the classification results showed that the 30 GO terms in which the candidate target genes were mainly involved are shown in Figure 3, including 10 terms each for biological processes (BP), cellular components (CC), and molecular functions (MF), as depicted in Figure 3. Within the BP category, the candidate genes were predominantly involved in small GTPase-mediated signal transduction (GO:0007264) and intracellular signal transduction (GO:0035556). Regarding CC, enrichment was observed in supramolecular complex (GO:0099080) and supramolecular polymer (GO:0099081). For MF, molecular function regulator (GO:0098772) and guanyl-nucleotide exchange factor activity (GO:0005085) were most represented, reflecting roles in transcriptional regulation, enzymatic activity modulation, organelle dynamics, and cellular stress responses.

**FIGURE 4:**
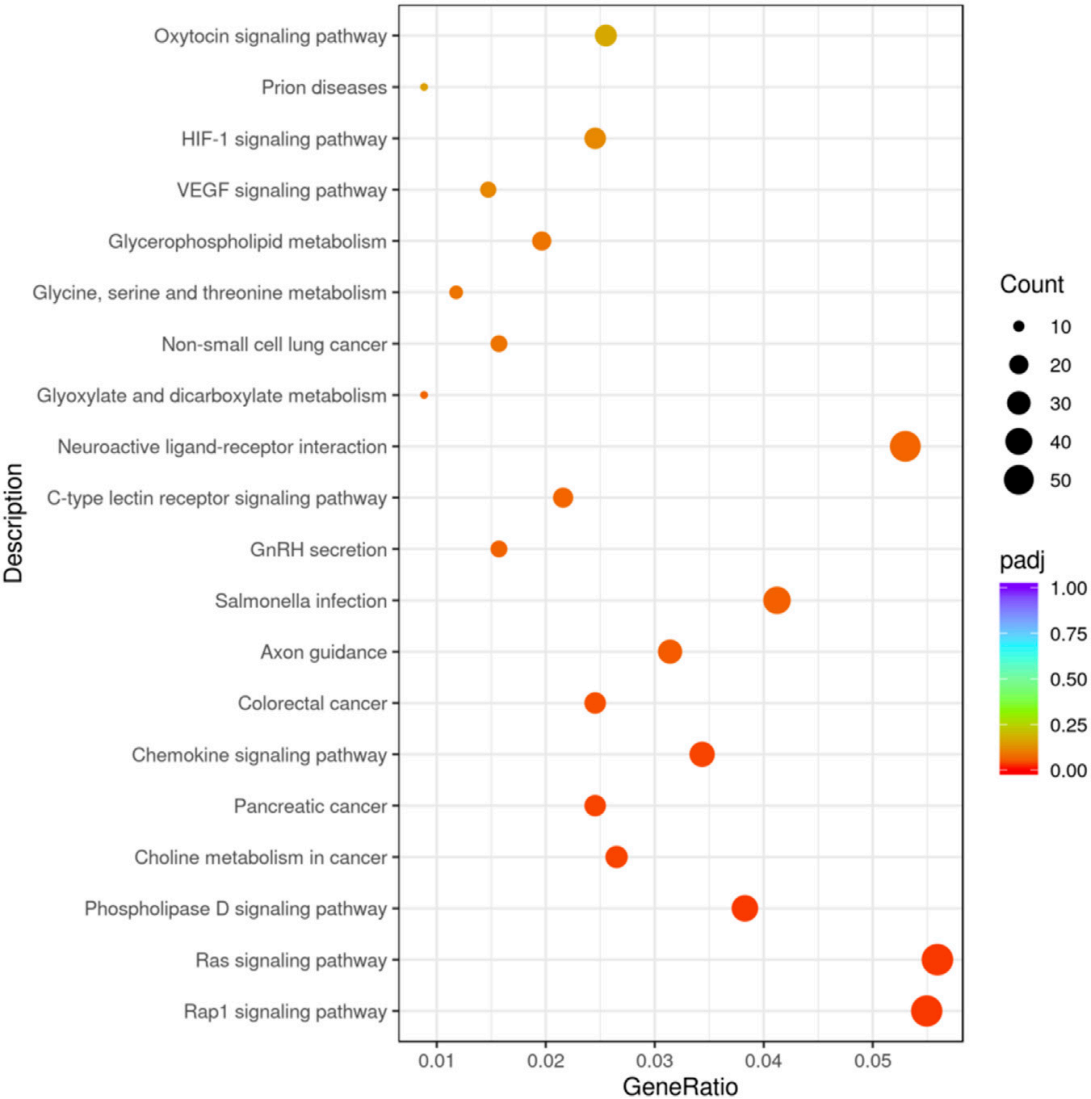
KEGG enrichment analysis Scatter plot.

The KEGG pathway covers a total of 117 target genes, and the results show that the candidate target genes are mainly involved in 20 pathways, combined with Figure 4, indicating that these candidate target genes are mainly involved in biological processes such as Rap1 signaling pathway, Ras signaling pathway, and Phospholipase D signaling pathway.

### 3.4 miRNA-qPCR assay

**FIGURE 5:**
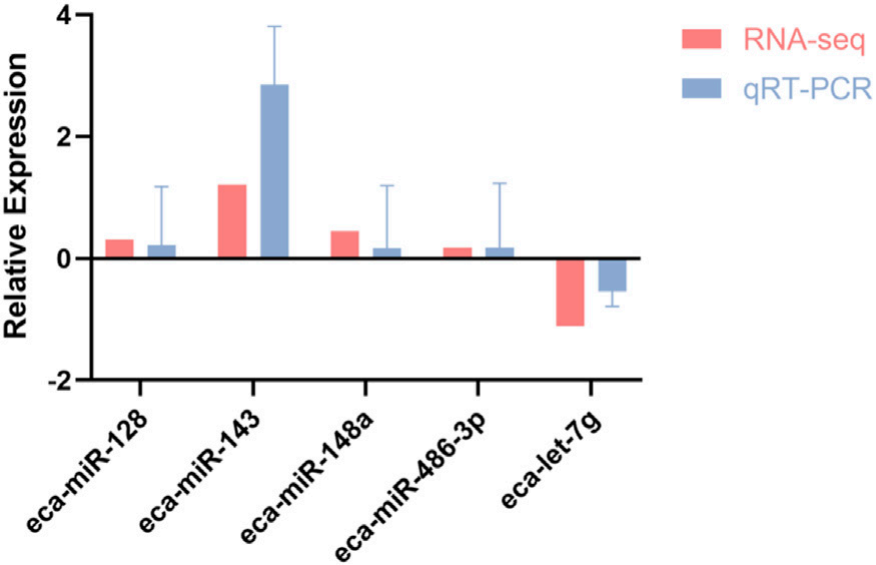
Correlation of RNA-seq and qRT-PCR expression profiles for five differentially expressed miRNAs in racing horses.

To evaluate the consistency between transcriptomic profiling and experimental validation, *miR-128*, *miR-143*, *miR-148a*, *miR-486-3p*, and *let-7g* were randomly selected for qRT-PCR analysis. As illustrated in Figure 5, expression patterns derived from qPCR closely mirrored those obtained from RNA-seq, thereby confirming the robustness of the sequencing dataset and reinforcing its reliability for further analyses.

## 4 Discussion

Exercise can induce gene expression modulation through epigenetic mechanisms, with miRNAs serving as key regulatory elements in this process (Grazioli et al., 2017). A total of 19 differentially expressed miRNAs were identified in this study. Existing research indicates that some of these are associated with exercise adaptation. However, their target genes and regulatory mechanisms remain computationally predicted and require experimental validation. Our bioinformatics analysis predicts multiple target genes that have been confirmed by previous studies to be strongly related to athletic performance and muscle adaptation.

Some researchers suggest that *miR-1* serves as a biomarker for aerobic activity, with its plasma levels significantly increasing after exercise (Andrea et al., 2019). This is primarily because during physical activity, mechanical overload stimulates skeletal muscles to secrete extracellular vesicles (EVs) enriched with *miR-1* (Vechetti et al., 2021). These EVs deliver *miR-1* to adipose tissue and promote lipolysis by targeting the *Adrb1* and *Hsl* genes, thereby providing energy for exercise. This process may facilitate the mobilization of energy substrates (such as free fatty acids), thus supporting prolonged physical activity and post-exercise recovery. The upregulation of plasma *miR-1* observed in Yili horses after racing in our study is consistent with this systemic adaptive mechanism. This may be mediated by its coordinated regulation of energy metabolism, particularly fatty acid metabolism, to enhance energy supply during racing. Similar results have been found in studies of human marathon runners, suggesting a possible association between *miR-1* and exercise (Eyileten et al., 2022; Chalchat et al., 2021). However, our findings lack more direct mechanistic confirmation.

Notably, *miR-486-3p* was predicted to target several key genes, including *CDKN1A* and *TMSB4X*. *CDKN1A*, implicated in muscle fiber composition, exhibits elevated expression following strength or sprint training (Semenova et al., 2022). *TMSB4X* functions as a movement- and growth-related factor and chemoattractant released in response to muscle injury, and its plasma levels increase irrespective of exercise modality (Gonzalez-Franquesa et al., 2021). These findings from other researchers are highly consistent with our own results, Upregulation of *miR-486-3p* may contribute to accelerated muscle fiber repair in Yili horses, mitigate micro-lesion accumulation, and support ongoing cytoskeletal remodeling (Fan, 2020). The findings from studies on endurance racehorses also support our conclusions (de Oliveira et al., 2021), suggesting that miR-486 may be associated with the exercise performance of Yili horses. Additionally, acute high-intensity exercise induces inflammatory responses in equine subjects, and sustained exposure may contribute to the development of degenerative joint conditions (MacNicol et al., 2018). *miR-128* suppresses the expression of its target gene *TAB2*, thereby attenuating *TAB2*-mediated activation of the NF-κB signaling pathway, which limits inflammation and prevents excessive immune activation (Ren et al., 2021). Therefore, the upregulation of *miR-128* in this study may contribute to post-exercise recovery in Yili horses, supporting physiological homeostasis and mitigating inflammatory damage. Furthermore, *miR-128* is associated with myogenic processes such as myoblast proliferation and differentiation (Grieb et al., 2023), which may explain why it is affected by exercise in Yili horses. However, further validation with larger sample sizes and more direct evidence is still required.

Under exercise stress, *miR-143* coordinately regulates cellular remodeling and inflammatory repair processes by targeting multiple genes, including *AKAP13* and *STAT4*. Specifically, *miR-143* modulates the AKAP13-mediated RhoA signaling pathway to optimize cytoskeletal reorganization and adaptive remodeling of myocytes (Langeberg and Scott, 2015), while simultaneously alleviating excessive post-exercise inflammatory responses by suppressing *STAT4* (Watfo et al., 2004). We hypothesize that post-exercise upregulation of *miR-143* in Yili horses may mediate the following physiological adaptations: these effects significantly enhance the structural integrity of muscle fibers and accelerate inflammation resolution and tissue repair, thereby collectively improving exercise endurance, improve muscle microcirculation, alleviate post-exercise ischemia, protect vascular endothelium from oxidative damage, and enhance osteoblast differentiation-thereby reducing the risk of stress fractures while improving bone elasticity, exercise tolerance, and recovery capacity.

High-intensity exercise imposes substantial energy demands on horses, predisposing them to rapid glycogen depletion, lactate accumulation, and metabolic dysregulation (Jose-Cunilleras, 2024). *miR-148a* suppress *PTEN* expression, thereby activating lipogenic signaling pathways such as *PPARγ* and *FASN*, ultimately promoting lipid deposition within intramuscular and subcutaneous adipocytes. This modulation of lipid storage is accompanied by enhanced insulin sensitivity and elevated glucose uptake (Jin et al., 2021), The upregulation of *miR-148a* may activate this coordination mechanism, attenuating lactate accumulation, delaying fatigue onset, and enhancing both racing and post-exercise recovery capacity in Yili horses.

*Let-7g* modulates glucose metabolism through interaction with the KRAS-PI3K-Rac1-Akt signaling axis, relieving suppression of KRAS-driven pathways and thereby enhancing mitochondrial OXPHOS and glycolytic activity (Hung et al., 2023), the downregulation of *let-7g* observed in our study may release suppression of this metabolic axis, potentially optimizing energy substrate turnover during high-intensity racing in Yili horses. The core differential miRNAs found in this study are closely associated with the exercise adaptation phenotype, and their feasibility as biomarkers is supported by other studies. These molecules can provide quantitative indicators for early breeding of Yili horses (Herkenhoff, 2025).

GO enrichment analysis in this study revealed that candidate target genes are primarily associated with CC (cellular components) and are highly enriched in terms related to cytoskeletal structure and mechanical strength. This suggests that differentially expressed miRNAs may mediate exercise adaptation in Yili horses by regulating cytoskeletal assembly and organization (Mandal et al., 2022). We therefore hypothesize that these miRNAs potentially enhance structural integrity, force transmission efficiency, and resistance to damage in muscle fibers through promoting cytoskeletal remodeling and reinforcement (Herkenhoff, 2025). This mechanism may constitute an important molecular basis for the exceptional racing observed in Yili horses.

KEGG enrichment analysis of predicted candidate target genes of differentially expressed miRNAs indicated significant involvement in signal transduction pathways. Members of the Ras superfamily, particularly Rap1, were implicated in enriched pathways such as the Rap1 and Ras signaling pathways. Activation of RhoGAP enhances the hydrolysis of RhoA-GTP to its inactive GDP-bound form, thereby attenuating ROCK-dependent myosin phosphorylation (Rajagopal et al., 2013; Heron-Milhavet and Djiane, 2022). Concurrently, Ras signaling promotes plasma membrane localization of glucose transporters and upregulates the expression of glycolytic enzymes via B-Raf/MEK/ERK activation (Vojtek and Der, 1998; Koh et al., 2002). Differentially expressed miRNAs may modulate these signaling pathways to improve muscle contraction efficiency, enhance mobilization and utilization of energy substrates during exercise, and reduce exercise-induced muscle damage in Yili horses.

In conclusion, the 19 differentially expressed miRNAs in Yili horses post-race are unlikely to function in isolation. Instead, they potentially form a coordinated regulatory network that synergistically governs the complex physiological adaptations to endurance exercise by co-targeting central signaling pathways and biological processes. Future studies, such as constructing a comprehensive miRNA interaction network or experimental validation using dual-luciferase assays, are warranted to decipher the precise nature of these interactions.

It must be acknowledged that in order to investigate the pre- and post-race miRNA transcriptome profiles of elite Yili horses while accounting for competition performance, only the top three winning horses were selected for this study. This limited sample size may introduce certain limitations to the accuracy and generalizability of the findings. Furthermore, since the enrichment analysis results were consistent with the regulatory pathways of the identified miRNAs, functional validation of target genes—such as knockdown or overexpression experiments—was not conducted. Additionally, this study included only male Yili horses, all 4 years of age, reflecting the typical age at which racehorses achieve competitive success. While this approach helped control for variations in age, sex, and breed, the influence of these factors warrants further investigation in future studies.

## 5 Conclusion

This study characterized the dynamic miRNA expression profile associated with exercise-induced stress in Yili horses during a 5000-m race, identifying specific miRNAs—such as *miR-1* and *miR-486-3p*—as potential biomarkers for breeding selection. Corresponding target genes and regulatory pathways involved in the physiological response to exercise were also delineated. Future efforts may focus on functional validation of key miRNAs to support the establishment of an miRNA-based early-stage breeding assessment framework.

## Data Availability

The data presented in the study are deposited in the National Center for Biotechnology Information (NCBI) repository, accession number PRJNA1303378.
